# Bioengineering ethics for the age of microphysiological systems

**DOI:** 10.3389/fbioe.2025.1497060

**Published:** 2025-03-20

**Authors:** Maxence Gaillard

**Affiliations:** ^1^ Centre for Medical Ethics, Department of Health and Society, Faculty of Medicine, University of Oslo, Oslo, Norway; ^2^ Institut Supérieur de Philosophie, UCLouvain, Louvain-la-Neuve, Belgium

**Keywords:** informed consent, moral status, precision medicine, biobanking, ethics-by-design

## Abstract

The development of microphysiological systems (MPS) is pushing ethical standards in biomedical research to a breaking point. This article argues that only a perspective drawing from engineering ethics will be able to address the new challenges raised by organoids and organs-on-chips. Extending progressively the scope of moral questioning, we discuss successively the following areas: i) individual consent: when cell lines are generated and human biomaterial is circulated and incorporated into biotechnologies whose life cycle will far exceed the scope envisioned by donors and manufacturers, the classic notion of informed consent becomes almost obsolete, or at least needs to be revisited. ii) Collective deliberation: MPSs raise many expectations for animal replacement and the advancement of precision and regenerative medicine. The management of these prospects by different stakeholders, and for everyone, is itself an ethical challenge at the interface of science and society. iii) Consideration of novel entities: some complex microphysiological systems may be endowed with a moral status in the near future, and this will have an impact on how researchers treat them and work with them.

## Introduction: MPS ethics as an ethics of bioengineering

Organoids and organs-on-chips are arguably new types of cell cultures, or, in other words, new entities that contemporary biotechnology has created. Both fields are flourishing (Shoji et al., 2023), with numerous prospects for promising applications. Although based on different strategies, organoids and organs-on-chips are sometimes subsumed under the general umbrella of microphysiological systems (Skardal, 2024) because these three-dimensional cellular models represent complex structures and functions typical of living organs.

With the novelty in biotechnology being acknowledged and praised, to what extent do these entities raise new questions at the societal and ethical level? Should the emergence of microphysiological systems (MPS) prompt us to reflect on or adapt the ethical and regulatory standards that have governed biomedical research until now?

To date, the main aspects of biomedical research ethics have been respect for donors and patients and general concerns about scientific integrity. These aspects are important as prerequisites for conducting research, but the implications of MPSs go far beyond these considerations. Consider, for instance, organoids and stem cell building blocks, that may originate from a variety of sources, such as established cell lines, supernumerary embryos, healthy donors, and patients. Human stem cell research is generally highly regulated, for instance by professional guidelines (ISSCR, 2021). The field has faced controversies from the beginning, especially because human embryo cells have been considered a controversial biomaterial. However, ethics extends beyond questioning the origin of stem cells, and they have also been scrutinized for their potential benefits to healthcare (Hyun, 2013).

The remarkable properties of these new objects in contemporary biology have fueled a discourse in which human beings are envisioned as taking control of biological processes that are considered ineluctable, such as aging. The development of induced pluripotent stem (iPS) cells is a hallmark of this shift into a new era—at least in the discourse surrounding biomedical research and innovation— that ultimately questions even our role in nature. Cells are the primary material that constitutes us as living bodies. They develop naturally in the body, following constraints such as differentiation and specialization. These processes appear to us as deterministic, and out of our control. Our ability to engineer complex *ex vivo* models from cells is changing this view. The ability of organoids to self-organize to the extent that cell cultures resemble organs is often acknowledged. However, although there are gray areas in their development, organoids are not natural organs that develop on their own; they are certainly highly controlled in the laboratory. They are cell cultures that replicate, reproduce, or mimic certain characteristics of organs. Organ-on-a-chip technology and microfluidics bring the possibility of emulating and monitoring a physiological environment outside the human body. In this regard, MPS are more than regular cell cultures and represent the culmination of the recent history of mastery over nature and life.

This is where MPS ethics should differ from existing frameworks. To account for the biotechnology revolution underway, a new perspective on ethics is needed. The ethics of bioengineering should focus on the purpose and methods of engineering and manipulating 3D cellular constructs. The ethics of engineering ask questions about why and how we build things. It starts with concerns such as is the X that I am about to build good for humankind? Could X be improved by adopting a different design? If implementing X seems like a good idea at first glance, are there any negative aspects or unintended consequences in the short or middle term, upon reflection? If so, do I have alternate design plans to avoid these undesirable consequences or make their occurrence less likely?

**FIGURE 1:**
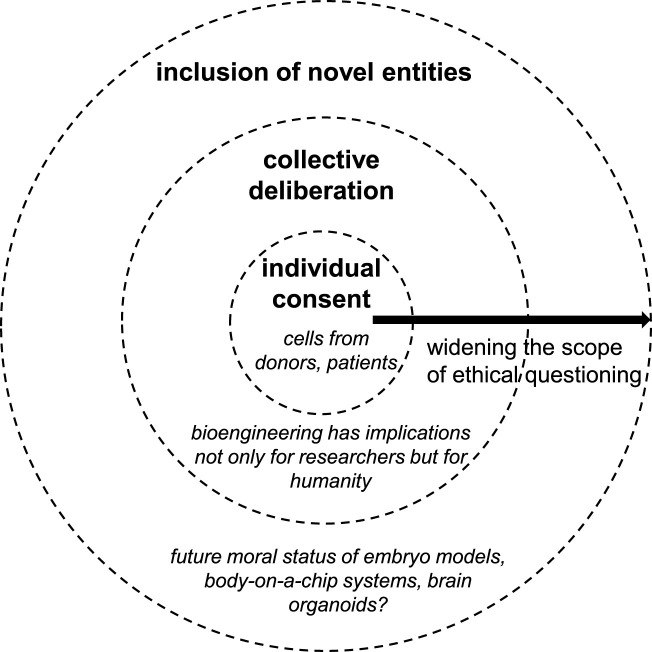
Three-stage model for global inclusion in MPS design.

Considering the moral significance of MPS and their potential impact on human nature, MPS design should be discussed globally with shared goals and benefits in mind. The discussion can be envisioned at three levels (Figure 1): the individual level, where individuals consent to researchers using their cells or biomaterials; the collective level, which encompasses society and humanity—beyond the stakeholders or direct actors in biotechnology research; and finally, an anticipatory scenario in which the moral community is extended to include some of the novel entities themselves.

## Individual: the future of informed consent for human cells in biotechnology

Although cell collection, storage, and use for research and clinical purposes are already regulated, new techniques provide a rationale for changing the ethical and legal framework. We should not forget that we are dealing with research objects—moving entities whose characteristics are likely to change in the years to come. Although it would be meaningless to change the rules for every new methodology, starting from the assumption that MPSs have new properties and thus deserve specific treatment, the mere existence of MPSs is a game changer in terms of the collection and use of cells.

Whether cells are used for research and testing immediately at the facility where samples are collected, transformed into cell lines that are available to other laboratories, possibly via commercial channels, or stored in biobanks for future use, they come from human donors whose wishes must be respected. Donors may object to certain applications. Informing donors and obtaining their consent are prerequisites for any action involving their cells. However, donors provide their consent based on the information available at the time, which reflects the technical possibilities of that period. Can we consider that donors who gave their consent to work on their cells based on a given zeitgeist (spirit of the time) automatically agree as techniques evolve?

This is not a matter of discarding consent forms for each new technique or application, as there are certain consent procedures that are intentionally broad or open-ended. From a donor’s perspective, developing a stomach or kidney organoid for basic research or drug screening is probably not fundamentally different from conducting similar research or screening drugs on 2D cell cultures. However, some donors may object to their cells being used in models that replicate certain organs, such as the brain, or in models that mix their cells with animal cells (chimeras). Donors willing to contribute to basic research may simultaneously reject the idea of integrating their cells into biotechnological constructs for transplantation into animals or even patients, thus limiting clinical applications such as regenerative medicine. For researchers, the shift in perspective toward bioengineering ethics means replacing a framework in which consent is obtained prior to technology development as a formal prerequisite with a continuous effort to respect donor intentions in the design of biotechnology and throughout the entire R&D pipeline.

Biobanking management, in particular, must find ways to align with the novelty that biotechnology can provide, ensuring that the technological possibilities of the time and the potential for new entities to be created are reflected in donor consent (Boers et al., 2019; Isasi et al., 2024). Some of the procedures under consideration include dynamic consent (where consent must be collected again before significantly new research is launched) or consent for governance (where a third party is entrusted by donors to make ethical decisions on their behalf). One has to find the right balance between respecting the will of donors by conveying exact information and enabling research that necessarily explores uncharted territory.

Managing the intrinsic uncertainty of future technological developments is not only a question of donor consent, but also relates to how we manage potential incidental findings (e.g., in cases where personalized *in vitro* models allow for new predictions). The possibility of the commodification of biotechnology and the embedding of human cells in complex biotechnological constructs is also connected to issues of patentability and fair distribution of potential benefits. As new MPS gain power in terms of individual prediction and clinical application, these issues will become more important, forcing us to revise existing rules to protect individual donors.

## Collective: including everyone and managing prospects on potential applications

Embracing a larger outlook, there is also a shift of perspective required at the collective level. Bioengineering ethics should consider the potential benefits and applications of MPS research and how we, as a collective—which includes both researchers and the public—relate to the expectations set by new biotechnologies (Ravn et al., 2023). Bioengineers often engage in a future-oriented discourse, highlighting the bright prospects of new technologies. These prospects, however, could be scrutinized and cannot be solely defined by the actors of biotechnology (e.g., researchers and technology developers, clinicians, and decision-makers). In other words, a collective perspective should be adopted from the beginning, ideally to include all those concerned in the design of bioengineered products.

The first prospect offered by MPS is the replacement of animal models in preclinical research, which not only offers tools that are more socially acceptable but also overcomes some of the well-known limitations of animal models for translational research, leading to predictions that are more relevant to human health at lower cost (Stresser et al., 2024). For clinical applications, one prospect is the significant advancement of functional precision medicine by screening the efficacy of different treatment options for individual patients (Beekman, 2016; Bose et al., 2021). Another clinical application is regenerative medicine, which could use MPS as a toolbox for improving tissue engineering and, ultimately, providing materials for restoring function, tissues, or organs (Xinaris, 2019).

These three prospects are different, and it is important to remember that any presentation of MPS research should state clearly what the prospects are and avoid confusion by combining promises that may not always be relevant to a specific research aim. They have, however, one thing in common: the ultimate goals (reducing animal suffering and improving and personalizing medicine) do not appear to be contentious at first glance. However, it is important to have a clear understanding of all the challenges that will arise, ranging from practical feasibility to regulatory aspects. Each prospect raises its own challenges, but they all face bottlenecks related to the difficulty of engineering MPS and standardizing techniques, the cost of the procedures, and the prerequisite for careful, transparent, and public documentation of each claimed success and failure. When the way forward is so clearly visible, there is a risk that the shared enthusiasm will tend to overlook epistemological challenges and bypass proper clinical trials. As a consequence, a focus on model validation (e.g., comparing animal and non-animal models), ensuring proper documentation of the mechanisms at play *in vitro*, and considering justice (e.g., representativeness of models and access to new treatments for all in need) is crucial. The ethics of bioengineering, as an effort to include all stakeholders, suggests that the concerns mentioned above are not just a result of research and development, but an integral part of biotechnology design. Validation, documentation, evaluation of prospects, and mitigation of risks are critical from the early stages of technology development to the final product.

## Novel entities: extending the moral community

From a broader perspective, technology may reach a critical stage where MPS must be progressively recognized as novel entities within the moral community. This would become necessary if some MPS were to attain moral status.

Philosophers define moral status as the idea that an entity inherently possesses certain rights due to its nature. Cell cultures do not possess a strong moral status: researchers must respect cell donors and their wishes, safeguard patients in clinical research, and treat animals used in research humanely. However, no specific moral consideration appears to apply to a collection of cells in a Petri dish. These cells are valuable for research or patient treatment, but not in themselves; their value is purely instrumental. These considerations also apply to the majority of MPS, such as intestinal, liver, or lung organoids. Consider, for example, a tumoroid or a tumor-on-a-chip derived from a patient’s cancer cells obtained through a biopsy or resection. These cells, which would otherwise be discarded, are cultured *in vitro* as a tumor model to test therapies. Ultimately, researchers or clinicians dispose of them like any other biological residue from a surgical operation or a laboratory test. However, we could argue that certain MPS are no longer mere products and must be treated accordingly.

This would be the case for embryo models that reproduce early stages of embryonic development. The very possibility of developing embryos from stem cells, such as iPS cells, raises considerable ethical issues. It may be tempting to dismiss this issue because of the nascent state of the technology, but even if the technology for developing proper embryos is not fully mastered—and may never be—the question of the moral status of these models remains urgent as existing landmarks, such as post-fertilization days of culture, are mostly irrelevant. When should an embryo model developed from stem cells be treated as an embryo? This question is difficult because the moral status of the embryo is controversial, and its legal definition varies depending on the jurisdiction. There are currently rules for research on embryos, but the extent to which these rules apply to stem cell-based embryo models is unclear, and consequently, these criteria are actively being discussed (Rivron et al., 2023). When the model comes close to being an embryo, it must be treated with care, in accordance with specific ethical and legal standards. In a sense, it is no longer just a research tool.

Complex physiological systems such as body-on-chips or human-on-chips may raise similar issues. In contrast to simpler *in vitro* models, these systems are useful as they provide insights into, for example, toxicity effects at the level of the whole organism. However, what degree of integration, self-regulation, or autonomy is required for interconnected cells to be considered an organism? Is the microenvironment a kind of “internal milieu” (Claude Bernard), and can the system be akin to a living being? What does it take to create artificial life? More specifically, at what point is this consideration likely to impact the moral status of these systems? The neighboring field of synthetic biology has already elicited debates along these lines, and those working in the field may want to proceed with caution. Developing a complex physiological system is one thing, but drawing implications on its humanness is another—not all bioengineers are Frankensteins (Shelley, 2017).

The consideration of moral status is also relevant when it comes to neural cells and brain tissue developed or maintained alive *ex vivo*. One of the criteria for attributing moral status to the creatures around us is sentience: we treat primates with more consideration than flies because we believe that they experience greater suffering from the treatment we inflict on them. Both are sentient beings, but the former has a more complex nervous system than the latter. If artificial nervous systems, such as brain organoids or brain-on-chips, were to develop a form of sensibility, pain, or even consciousness—the terms here are delicate and debated—they would *ipso facto* deserve a moral status in their own right and would, therefore, no longer be mere tools for unrestricted use (Pichl et al., 2023). This does not mean that researchers could not use them for research—after all, sentient beings (animals) are already used in laboratories—but such use would require a precise assessment of the capacities of these entities and an estimation of the costs and expected benefits in terms of wellbeing. This necessitates innovative methods, along with both epistemological and conceptual reflection on the nature of consciousness, and a careful consideration of the engineering method. The scenario may be hypothetical, but the possibility of considering the object of tissue engineering as a subject of rights represents a radical change in perspective—one that should be anticipated.

## Data Availability

The original contributions presented in the study are included in the article/Supplementary Material; further inquiries can be directed to the corresponding author.
